# Fenofibrate administration to arthritic rats increases adiponectin and leptin and prevents oxidative muscle wasting

**DOI:** 10.1530/EC-12-0003

**Published:** 2012-06-08

**Authors:** Estíbaliz Castillero, Ana Isabel Martín, Maria Paz Nieto-Bona, Carmen Fernández-Galaz, María López-Menduiña, María Ángeles Villanúa, Asunción López-Calderón

**Affiliations:** Department of Physiology, Faculty of Medicine Complutense University of Madrid 28040, Madrid Spain; Department of Histology, Faculty of Health Sciences Rey Juan Carlos University 28922 Alcorcón, Madrid Spain

**Keywords:** adjuvant-induced arthritis, oxidative muscle, adiponectin, leptin, PPAR alpha, fenofibrate, MyoD, myogenin, myostatin, Murf1, Insulin

## Abstract

Chronic inflammation induces skeletal muscle wasting and cachexia. In arthritic rats, fenofibrate, a peroxisome proliferator-activated receptor α (PPARα (PPARA)) agonist, reduces wasting of gastrocnemius, a predominantly glycolytic muscle, by decreasing atrogenes and myostatin. Considering that fenofibrate increases fatty acid oxidation, the aim of this study was to elucidate whether fenofibrate is able to prevent the effect of arthritis on serum adipokines and on soleus, a type I muscle in which oxidative metabolism is the dominant source of energy. Arthritis was induced by injection of Freund's adjuvant. Four days after the injection, control and arthritic rats were gavaged daily with fenofibrate (300 mg/kg bw) or vehicle over 12 days. Arthritis decreased serum leptin, adiponectin, and insulin (*P*<0.01) but not resistin levels. In arthritic rats, fenofibrate administration increased serum concentrations of leptin and adiponectin. Arthritis decreased soleus weight, cross-sectional area, fiber size, and its *Ppar* *α* mRNA expression. In arthritic rats, fenofibrate increased soleus weight, fiber size, and *Ppar* *α* expression and prevented the increase in *Murf1* mRNA. Fenofibrate decreased myostatin, whereas it increased *MyoD* (*Myod1*) and myogenin expressions in the soleus of control and arthritic rats. These data suggest that in oxidative muscle, fenofibrate treatment is able to prevent arthritis-induced muscle wasting by decreasing *Murf1* and myostatin expression and also by increasing the myogenic regulatory factors, MyoD and myogenin. Taking into account the beneficial action of adiponectin on muscle wasting and the correlation between adiponectin and soleus mass, part of the anticachectic action of fenofibrate may be mediated through stimulation of adiponectin secretion.

## Introduction

Cachexia is a multifaceted syndrome whose etiology is complex and is directly related to poor patient prognosis and survival (1). Chronic inflammatory illnesses such as cancer, sepsis, rheumatoid arthritis (RA), and chronic obstructive pulmonary disease are associated with a decrease in body weight and cachexia (2). Adjuvant-induced arthritis is an experimental model of RA that is induced in rats by an intradermal injection of Freund's adjuvant. Ten days after the injection, rats develop polyarthritis together with a marked loss of white adipose tissue (WAT) and skeletal muscle mass and cachexia (3, 4). Skeletal muscle wasting is not secondary to the decrease in food intake in arthritic rats, as it was not observed in pair-fed rats (4). In RA patients, dietary intake is not considered to play an important role in causing cachexia, but it instead seems to be determined by a combination of intensity, duration, and frequency of active inflammatory disease (5). In addition to inflammatory mediators, dysregulation of metabolism is an important contributor to inflammatory cachexia (2).

Peroxisome proliferator-activated receptors (PPARs) are a family of nuclear receptors that modulate metabolism and inflammation. PPARα (PPARA) agonists have been proposed as potential treatment for RA, due to their anti-inflammatory properties (6, 7). Okamoto & Kamatani (8) first reported anti-inflammatory effects of PPARα agonists when used clinically as treatment for dyslipidemia in RA patients. An anti-inflammatory effect of PPAR activation in rheumatoid synovials fibroblast cultures as well as in experimental models of arthritis has been reported (6). Furthermore, PPARα agonists are able to counteract the inflammatory and destructive action of interleukin 1 β (IL1B) in human osteoarthritic cartilage samples (9) and in rabbit articular chondrocytes (10).

Our group has reported that chronic arthritis decreases *Pparα* expression in liver and gastrocnemius, whereas the PPARα agonist fenofibrate improves arthritis-induced body weight loss and gastrocnemius muscle wasting by decreasing atrogenes and myostatin (11). Fibrates, as well as other PPARα agonists, regulate the expression of genes critical for lipid and lipoprotein metabolism. Adipose tissue is not only an energy storage organ but also an important endocrine organ secreting proteins known as adipokines. Adiponectin and leptin are adipocytokines secreted specifically by adipose cells and both may play an important role in chronic inflammation and autoimmune diseases (12). Adjuvant-induced arthritis in rats decreases WAT mass, serum leptin, and adiponectin as well as its expression in the adipose tissue (3, 13). In addition, muscles of adiponectin-deficient mice exhibit a higher degree of oxidative stress and apoptosis than those of wild-type mice when challenged by lipopolysaccharide, and these abnormalities may be corrected by local administration of adiponectin (14). Taking into account that activation of PPARα by fenofibrate treatment increases serum adiponectin and resistin while it improves insulin sensitivity (15), the effect of fenofibrate treatment on adipokines in arthritic rats was studied in this study.

Skeletal muscle is made up of two main types of fibers. Type I or oxidative, such as soleus, that produces ATP from cellular respiration. Type II, such as gastrocnemius, has fewer mitochondria and its ATP production is mainly dependent on glycolysis. Both types of muscles are wasted in arthritic rats, although gastrocnemius to a greater extent than soleus (16). Increased fatty acid uptake and β-oxidation are clinical benefits of fenofibrate. Therefore, the effect of fenofibrate administration on soleus muscle was also analyzed. For this purpose, we analyzed the effect of fenofibrate on morphology and *ATROGIN1* (*Fbxo32*), *Murf1*, myostatin, *MyoD* (*Myod1*), and myogenin expressions in soleus of control and arthritic rats.

Our data showed that fenofibrate increased circulating adiponectin and leptin and prevented soleus wasting in arthritic rats, where adiponectin correlated with soleus mass. In addition to decreased *Murf1* and myostatin in soleus, fenofibrate increased *MyoD* and myogenin expressions.

## Materials and methods

Arthritic and control male Wistar rats (150 g/6 weeks old) were purchased from Charles River Laboratories (Barcelona, Spain). Arthritis was induced in the rats by an intradermal injection of 4 mg heat-inactivated *Mycobacterium butyricum* in the right paw under isoflurane anesthesia. Control animals were injected with vehicle (0.1 ml paraffin oil). After arriving at our facilities (day 3 after adjuvant injection), three to four rats were housed per cage and maintained under standardized conditions of temperature (20–22 °C) and light (lights on from 0730 to 1930 h). Water and standard chow (A=04; Panlab, Barcelona, Spain) were provided *ad libitum*. The procedures were followed according to the guidelines recommended by the EU for the care and use of laboratory animals and were approved by the Complutense University Animal Care Committee.

On day 4 after adjuvant injection, both control and rats injected with adjuvant were randomly divided in two groups of 34 rats. The first group received fenofibrate (300 mg/kg bw, suspended in 500 μl of 1% carboxymethyl cellulose (CMC); Sigma–Aldrich) daily by oral gavage. The second group was gavaged with vehicle (500 μl 1% CMC). Pair-fed rats (*n*=17) received the same amount of food (g/100 g bw) consumed by arthritic rats treated with vehicle on the previous day and were gavaged daily with vehicle. Arthritis severity was evaluated by measuring the arthritis index of each animal, which was clinically scored by grading each paw from 0 to 4, as inflammation of the paw is associated with radiological and histological alterations of the joints. Grading was determined as follows: 0 – no erythema or swelling; 1 – slight erythema or swelling of one or more digits; 2 – swelling of paw; 3 – swelling of entire paw and the ankle; and 4 – ankylosis, incapacity to bend the ankle. The severity score was the sum of the clinical scores of the four limbs, the maximum value being 16. After 12 days of fenofibrate treatment, and 15 days after adjuvant or vehicle injection, all rats were killed by decapitation between 1200 and 1300 h, in a separate room, within 30 s after being removed from their cages. Trunk blood was collected in cooled tubes, allowed to clot, centrifuged, and the serum was stored at −20 °C until adiponectin, leptin, resistin, insulin, and glucose assays were performed. Liver was removed, dissected, frozen, and stored at −80 °C until glycogen analysis. Left gastrocnemius and periepididymal WATs were dissected and weighed. Left soleus from nine rats of each group were dissected, weighed, frozen in liquid nitrogen, and stored at −80 °C until RNA or protein extraction. Isolation and manipulation of tissues were always performed under sterile conditions.

### Soleus morphology

Left soleus from eight rats per group was used for immunohistochemical study. Muscles were dissected, weighed, placed on a transparency film, glued at one end to a cork with gum tragacanth (Fibraguar, Fardi, Madrid, Spain), frozen in isopentane, cooled by liquid nitrogen, and stored at −80 °C. Cryostat sections (10 μm) were fixed with 100% acetone and stained with hematoxylin–eosin. Parallel sections were kept at −80 °C until further processing for immunohistochemical analysis. Four to six muscle hematoxylin–eosin-stained sections were used to determine the whole cross-sectional area. Sections were scanned (Epson scanner 4990) with a transparent rule and the area was measured using Image J software (Bethesda, MD, USA).

Muscle fiber cross-sectional size was as an index of fiber atrophy. The extracellular matrix was detected by Wheat Germ Agglutinin (WGA) labeled with Texas Red (W849; Invitrogen; 1 μg/ml). Sections were mounted with Prolong-Gold antifade reagent combined with DAPI (P36931; Invitrogen). Digital images were acquired using a Leica DMI300 microscope. Fiber boundaries were detected from the WGA fluorescent images using Difference of Gaussians algorithm by GIMP software (Groton, MD, USA). At least two images from each section were used to measure the fiber minimal feret diameter using ImageJ software.

### RNA extraction and real-time PCR

Solei were homogenized, and total RNA was extracted using Ultraspec (Biotecx Laboratories, Inc., Houston, TX, USA) following the manufacturer's protocol. The final concentration of RNA was determined (260 nm) using a BioPhotometer (Eppendorf, Germany), and the integrity of the RNA was confirmed by agarose gel electrophoresis. First-strand cDNA synthesis was performed using 1 mg total RNA with a Quantiscript Reverse Transcription kit (Qiagen).

Real-time PCR for quantification of mRNA was performed on a SmartCycler (Cepheid, Sunnyvale, CA, USA) using a SYBR-Green protocol in the fluorescence temperature cycler. Each real-time PCR consisted of 10 ng total RNA equivalents, 1× Takara SYBR Green Premix Ex Taq (Takara BIO, Inc., Otsu, Shiga, Japan), and 300 nM forward and reverse primers in a reaction volume of 25.5 μl. Primers for real-time PCR were obtained from Roche using the EXIQON Universal Probe Library (Table 1). The thermal cycling profile consisted of a preincubation step at 95 °C for 10 s followed by 40 cycles of 95 °C denaturation steps for 15 s, 60 °C annealing steps for 30 s, and 72 °C extension steps for 30 s. Results were expressed as fold changes in expression of each gene in arthritic and pair-fed rats compared with control animals treated with vehicle using cycle threshold 2(ΔΔ*C*_T_) method with *18s* as reference gene.

### Western blot

Soleus samples were homogenized in 1 ml lysis buffer with protease inhibitor cocktail (Sigma–Aldrich). The homogenate was later centrifuged at 13 226 ***g*** at 4 °C for 30 min to remove tissue debris. Protein concentration was determined using the Bradford protein assay with BSA as standard. The protein extract was boiled for 5 min with a 1:1 volume of Laemmli loading buffer. Proteins (100 μg) were resolved by electrophoresis on 14% polyacrylamide gels under reducing conditions and then transferred onto nitrocellulose membranes that were blocked by incubation in 5% nonfat dry milk, 0.1% Tween (Sigma–Aldrich), and in Tris-buffered saline. Membranes were probed overnight at 4 °C, sequentially with antibodies against myostatin, MyoD, and myogenin (Santa Cruz Biotechnology, Santa Cruz, CA, USA) and α-tubulin (Sigma–Aldrich) with stripping of membranes before each new antibody. Membranes were then incubated for 90 min in the appropriate secondary antibody conjugated to HRP (antimouse IgG, Amersham Biosciences; antirabbit IgG, Bio-Rad), and peroxidase activity was detected using enhanced chemiluminescent reagent (Amersham Biosciences). Band intensities were quantified by densitometry using a PC-Image VGA24 program for Windows. The density of the protein band in each lane was expressed as the percentage of the mean density of control rats, after load normalization using α-tubulin.

### Serum measurements

Serum concentrations of rat adiponectin, leptin, and insulin were determined by RIA using commercial kits from Millipore (Billerica, MA, USA). Rat serum resistin was determined by ELISA from Biovendor GmbH (Heidelberg, Germany). Serum glucose was measured using a colorimetric kit from Cayman (Ann Arbor, MI, USA).

### Glycogen

Glycogen was extracted from liver samples by boiling them in 30% KOH and neutralizing with Na_2_SO_4_. Extracts were deproteinized with trichloroacetic acid and glycogen was precipitated in ethanol and then subjected to 6 M HCl followed by neutralization. The resulting concentration of glycosyl residues was measured using the glucose determination kit.

### Statistical analysis

Results were compared using the statistics program STATGRAPHICS plus for Windows. Data are presented as mean±s.e.m. Statistical evaluation of the data was performed by one-way ANOVA; *post hoc* comparisons were made using the LSD multiple range test. Statistical significance was set at *P*<0.05.

## Results

Fenofibrate treatment over 12 days decreased the external signs of inflammation from 11±0.69 (arthritis scores±s.e.m.) in the arthritic rats treated with vehicle to 5.83±0.78 (*P*<0.01) in the rats treated with fenofibrate. Both arthritis and pair-feeding the rats had decreased body weight gain (Table 2; *P*<0.01), but pair-fed rats had higher body weight gain than arthritic rats treated with vehicle (*P*<0.01). Fenofibrate administration did not modify body weight gain in control rats, whereas it increased body weight gain in arthritic rats to levels similar to those of pair-fed rats.

Arthritis decreased epididymal WAT weight (*P*<0.01; Table 2). Similar to body weight gain, the decrease in epididymal WAT weight in arthritic rats can be secondary to inflammation and to the decrease in food intake, as pair-fed rats had epididymal WAT weight values between those of control and arthritic rats. Fenofibrate administration was not able to significantly increase absolute epididymal WAT weight in arthritic rats. Pair-fed rats had lower absolute gastrocnemius weight than control rats (*P*<0.01; Table 2), but relative gastrocnemius weight was similar in pair-fed and in control rats (483±20 mg/100 g bw ±s.e.m. vs 505±20). Arthritis decreased gastrocnemius and soleus weights (*P*<0.01). As we have previously reported (17), the inhibitory effect of arthritis on muscle mass is higher in gastrocnemius than in soleus (49% of control rats vs 58%). In arthritic rats, fenofibrate increased gastrocnemius weight to higher levels than arthritic rats treated with vehicle (*P*<0.01), but lower than those of pair-fed rats or control rats treated with fenofibrate (*P*<0.01). In contrast, fenofibrate prevented the inhibitory effect of arthritis on soleus weight, and arthritic rats treated with fenofibrate had similar soleus weight than pair-fed or control rats.

Arthritis decreased glycogen concentration in the liver (*P*<0.01; Table 2). This decrease seems to be due to inflammation, as pair-fed and control rats had similar liver glycogen. In control rats, fenofibrate decreased liver glycogen, but to a lesser extent than arthritis did. Serum concentration of glucose was not significantly modified by arthritis or fenofibrate treatment.

### Adipokines and insulin

**Figure 1:**
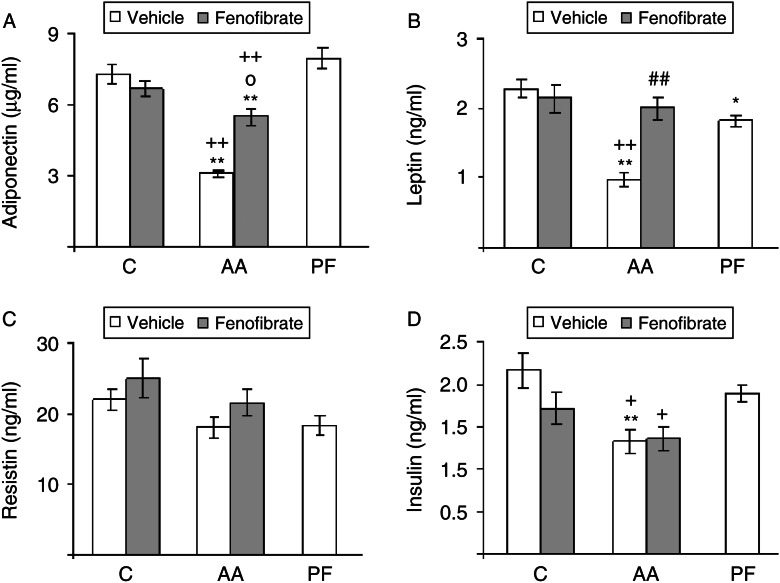
Serum concentrations of adiponectin (A), leptin (B), resistin (C), and insulin (D) in control (C), arthritic (AA), and pair-fed (PF) rats treated with fenofibrate (300 mg/kg) or vehicle. Arthritis decreased serum adiponectin, leptin, and insulin (*P*<0.01). In arthritic rats, fenofibrate increased serum adiponectin and leptin. Data represent mean±s.e.m. (*n*=9–17 rats). **P<0.01 vs control rats, *P<0.05 vs control rats, ^++^ *P*<0.01, ^+^ *P*<0.05 vs pair-fed rats, ^##^ *P*<0.01 vs arthritic rats treated with vehicle, °*P*<0.05 vs control rats treated with fenofibrate.

Arthritis decreased serum concentrations of adiponectin (*P*<0.01; Fig. 1A). This effect was not mediated by the decrease in food intake, as pair-fed rats had serum concentrations of adiponectin similar to those of the control rats. Fenofibrate administration to arthritic rats increased serum concentrations of adiponectin (*P*<0.01). Arthritis also decreased serum concentration of leptin (*P*<0.01; Fig. 1B). This decrease is in part due to the decrease in food intake, as pair-fed rats also had lower leptin levels than those of control rats (*P*<0.05). Fenofibrate treatment prevented the inhibitory effect of arthritis on serum leptin, whereas in control rats, fenofibrate did not modify serum concentration of leptin. In contrast to serum adiponectin and leptin concentrations, arthritis, fenofibrate treatment, or pair-feeding the rats did not change serum concentration of resistin (Fig. 1C).

Arthritis also decreased serum concentrations of insulin (Fig. 1D). This decrease is not secondary to modifications in food intake, as pair-fed rats and control rats had similar serum insulin levels. In control and in arthritic rats, fenofibrate treatment did not modify serum concentrations of insulin.

In arthritic rats, either treated with vehicle or fenofibrate, there was a positive correlation between soleus weight and circulating adiponectin (*r*=0.403, *P*<0.01). In contrast, there was not a correlation between soleus weight and serum concentrations of leptin, resistin, or insulin.

### Soleus

**Figure 2:**
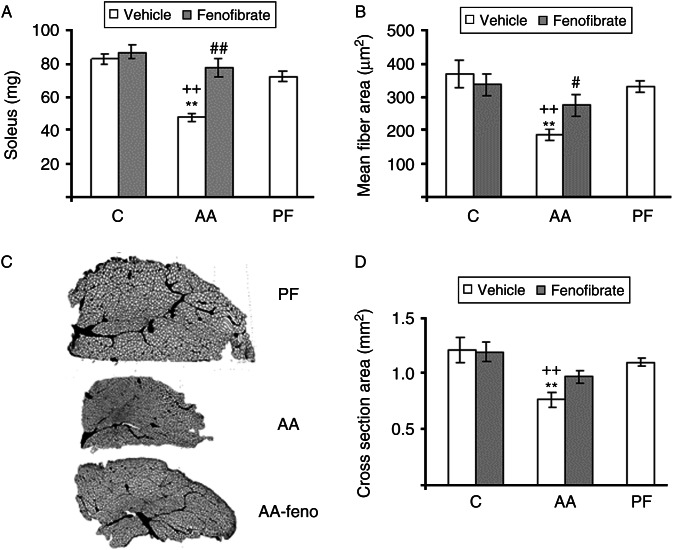
Effect of arthritis and fenofibrate (300 mg/kg) on soleus weight (A), mean fiber area (B), and cross-sectional area (D). Representative cross sections of the soleus (C). C, control; AA, arthritic, or PF, pair-fed. Arthritis decreased soleus weight, mean fiber area, and cross-sectional area (*P*<0.01). Fenofibrate increased soleus weight and fiber area in arthritic rats. Data represent mean±s.e.m. (*n*=6–8). **P<0.01 vs control rats, ^++^ *P*<0.01 vs pair-fed rats, ^##^ *P*<0.01, ^#^ *P*<0.05 vs arthritic rats treated with vehicle.

In addition to soleus weight, arthritis also decreased soleus cross-sectional area and mean fiber area (*P*<0.01; Fig. 2A, B, C and D). Those inhibitory effects are not secondary to the decrease in food intake, as they were not observed in pair-fed rats. Fenofibrate administration to arthritic rats increased mean fiber area (*P*<0.05; Fig. 2B). In arthritic rats treated with fenofibrate, soleus cross-sectional area values were between those of arthritic rats treated with vehicle and those of pair-fed rats (Fig. 2C and D).

**Figure 3:**
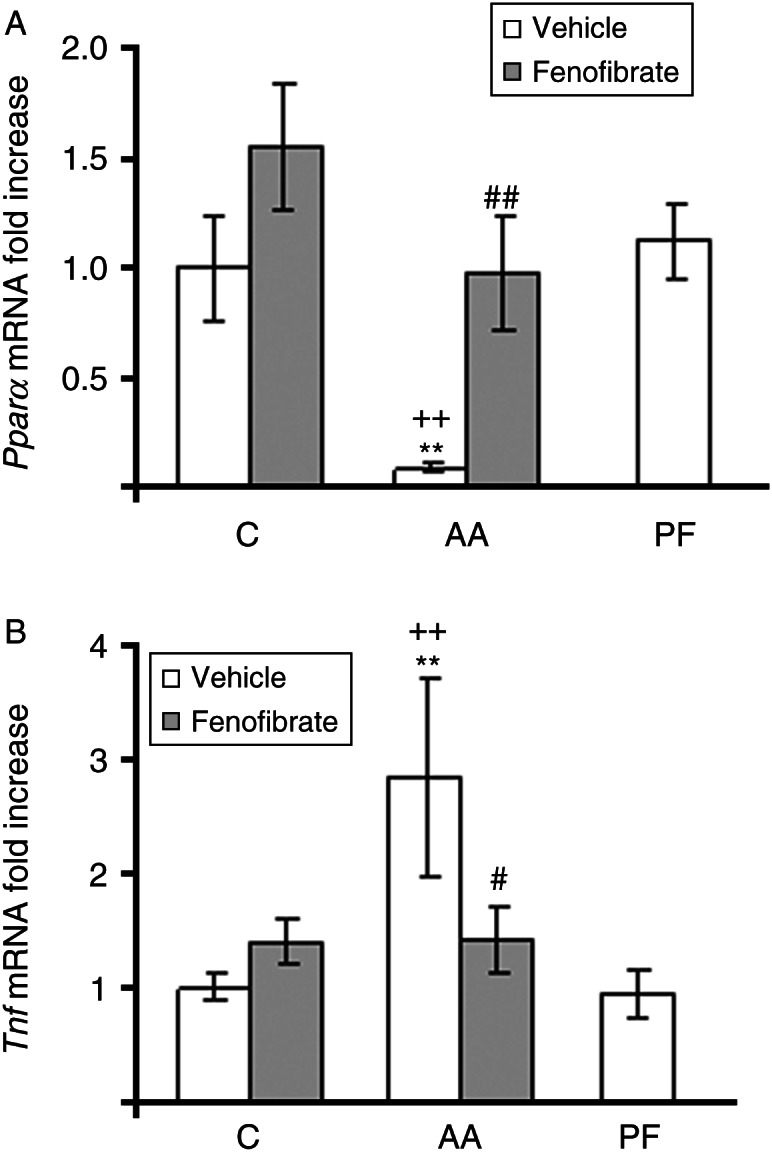
Effect of arthritis and fenofibrate (300 mg/kg) on *Pparα* (A) and *Tnf* (B) mRNA in soleus. AA, arthritic rats; PF, pair-fed rats. mRNAs were quantified using real-time PCR and are presented in relation to the mean value in control group. Arthritic rats have lower soleus *Pparα* and higher *Tnf* than control rats. Fenofibrate treatment to arthritic rats prevented both changes. Data represent mean±s.e.m. (*n*=6–9 rats). **P<0.01 vs control rats, ^##^ *P*<0.01, ^#^ *P*<0.05 vs arthritic rats treated with vehicle, ^++^ *P*<0.01 vs pair-fed rats.

*Pparα* expression in the soleus was dramatically decreased by arthritis in the rats treated with vehicle to 8.5% of controls (*P*<0.01) but not in those treated with fenofibrate (Fig. 3A). Pair-fed rats and control rats treated with fenofibrate had normal soleus *Pparα* mRNA levels. *Tnf* mRNA levels were increased in arthritic rats treated with vehicle (*P*<0.01), whereas in rats treated with fenofibrate, arthritis was not able to increase *Tnf* mRNA in soleus (Fig. 3B).

**Figure 4:**
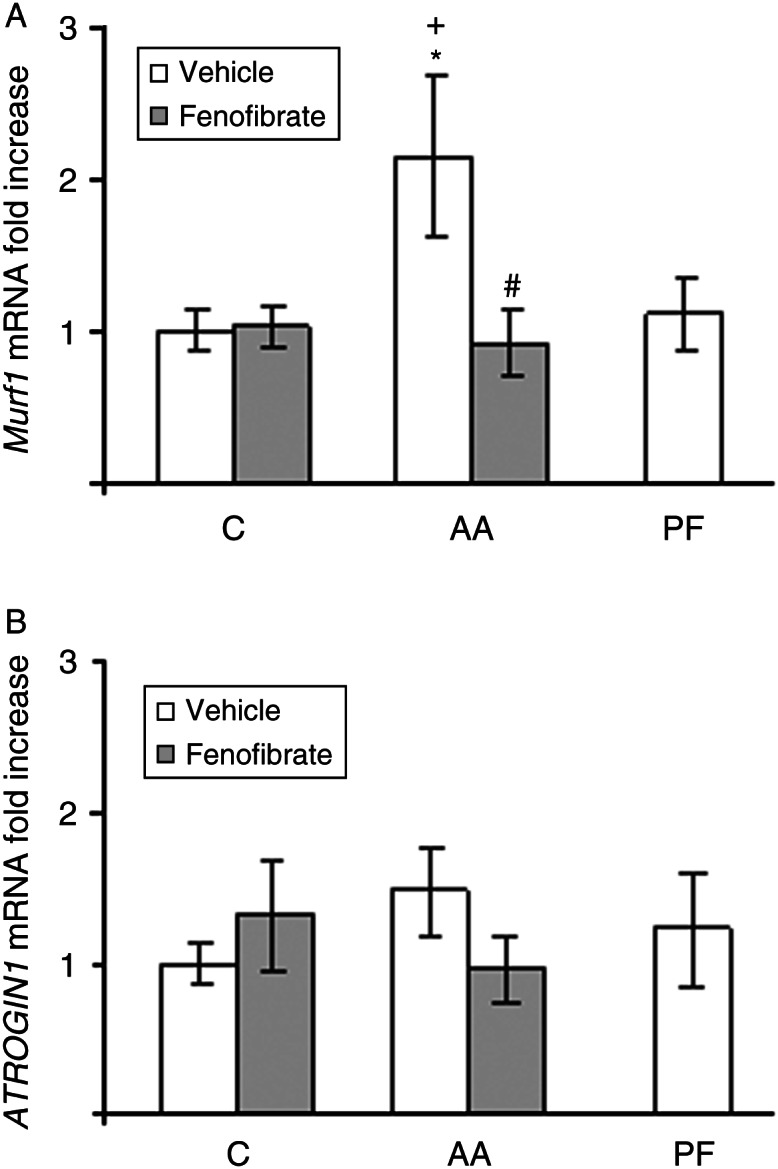
Effect of arthritis and fenofibrate (300 mg/kg) on *Murf1* (A) and *ATROGIN1* (B) mRNA in soleus. AA, arthritic rats; PF, pair-fed rats. mRNAs were quantified using real-time PCR and are presented in relation to the mean value in control group. Arthritic rats have higher *Murf1* than control rats. Fenofibrate treatment to arthritic rats prevented this increase. Data represent mean±s.e.m. (*n*=6–9 rats). *P<0.05 vs control rats, ^#^ *P*<0.05 vs arthritic rats treated with vehicle, ^+^ *P*<0.05 vs pair-fed rats.

Arthritis increased *Murf1* mRNA expression (*P*<0.05; Fig. 4A). In arthritic rats, fenofibrate treatment decreased *Murf1* mRNA levels (*P*<0.01). Arthritis, pair-feeding the rats, and fenofibrate treatment did not modify *ATROGIN1* expression in soleus muscle (Fig. 3B).

**Figure 5:**
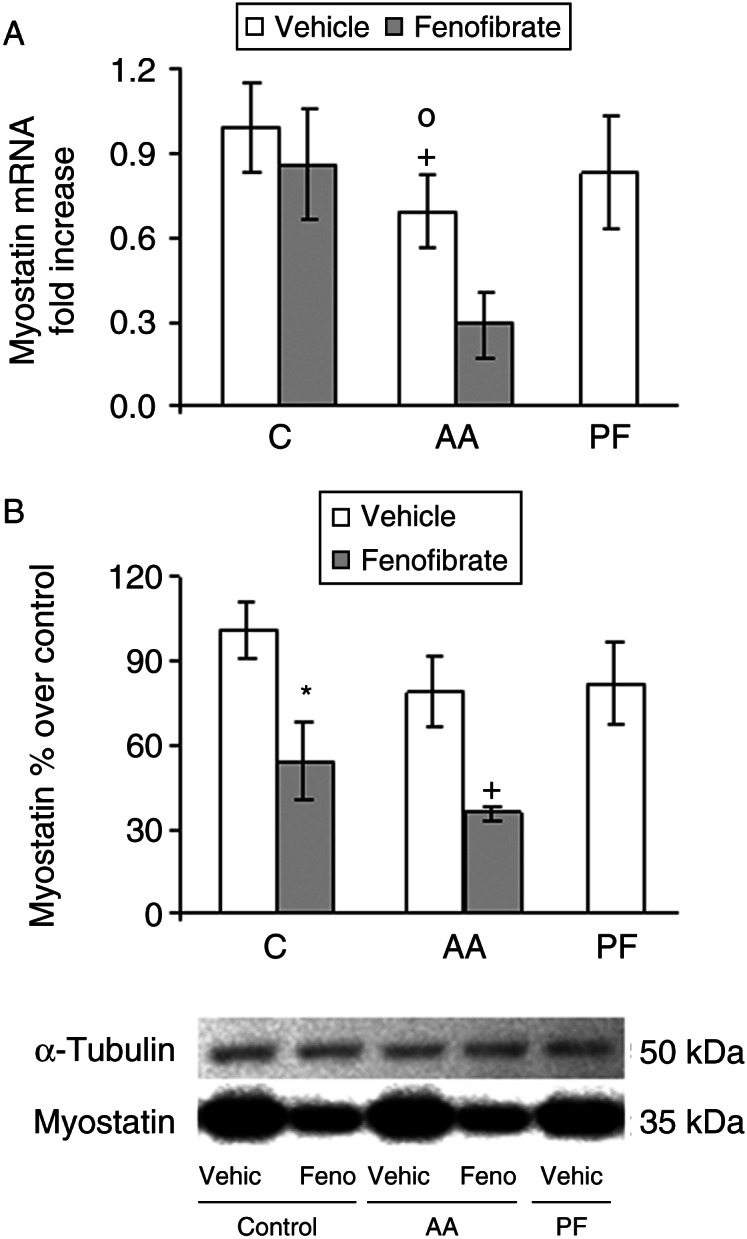
Effect of arthritis and fenofibrate (300 mg/kg) on myostatin mRNA (A) and protein (B) in soleus. AA, arthritic rats; PF, pair-fed rats. mRNAs were quantified using real-time PCR and are presented in relation to the mean value in control group. Myostatin was measured by western blot, quantified, normalized against the α-tubulin, and expressed as percentage of the control rats. Fenofibrate decreased myostatin mRNA and protein. Data represent mean±s.e.m. (*n*=6–9 rats). *P<0.05 vs control rats, ^+^ *P*<0.05 vs pair-fed rats, °*P*<0.05 vs control rats treated with fenofibrate.

Myostatin mRNA and protein content were not modified by arthritis (Fig. 5A and B). Arthritic rats treated with fenofibrate had lower myostatin mRNA and protein levels than pair-fed rats (*P*<0.05). In control rats, fenofibrate administration decreased myostatin protein content (*P*<0.05).

**Figure 6:**
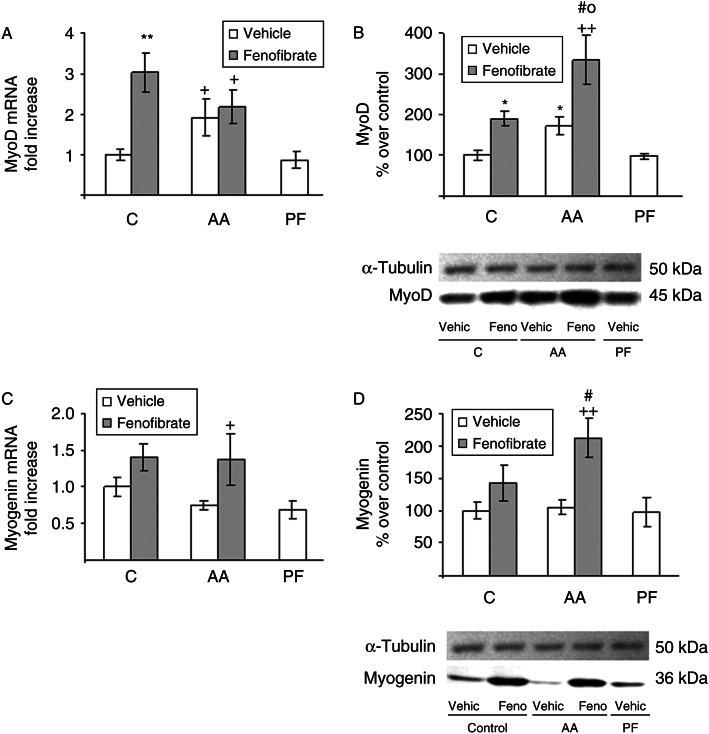
Effect of arthritis and fenofibrate treatment (300 mg/kg) on soleus MyoD (A and B) and myogenin (C and D) mRNA and protein. mRNAs were measured by real-time PCR and presented in relation to the mean value in control group. Proteins were measured by western blot, normalized against the α-tubulin, and expressed as percentage of the control rats. Arthritis and fenofibrate increased MyoD mRNA and protein. Fenofibrate increased myogenin mRNA and protein in arthritic rats. Data represent mean±s.e.m. (*n*=5–9 rats). **P<0.01, *P<0.05 vs control rats, ^#^ *P*<0.05 vs arthritic rats treated with vehicle, ^++^ *P*<0.01, ^+^ *P*<0.05 vs pair-fed rats, °*P*<0.05 vs control rats treated with fenofibrate.

As shown in Fig. 6A and B, arthritic rats had higher soleus *MyoD* mRNA and protein in comparison with pair-fed and control rats (*P*<0.05), whereas myogenin expression was not modified by arthritis (Fig. 6C and D). Pair-feeding the rats did not change MyoD or myogenin. Fenofibrate administration increased the expression of both myogenic regulatory factors. MyoD mRNA and protein were upregulated by fenofibrate treatment in both control and arthritic rats. However, fenofibrate treatment increased myogenin mRNA and protein levels only in arthritic rats.

## Discussion

Our data show that fenofibrate administration ameliorates the inhibitory effect of arthritis on serum leptin and adiponectin. In addition, fenofibrate reverts arthritis-induced decrease in soleus mass and this effect is associated with decreased expression of *Murf1* and myostatin, as well as upregulation of MyoD and myogenin expressions.

As previously reported (3, 13, 18, 19), arthritis induced a marked decrease in serum concentrations of adiponectin and leptin, whilst serum resistin was not significantly decreased. Similar data have been reported in mice with cancer cachexia, in which severe weight loss is associated with decreased leptin and insulin, whereas resistin remains unchanged (20). However, most studies on RA patients reported that leptin, adiponectin, and resistin levels are increased in serum and synovial fluid in comparison with healthy control subjects (for review, see (21, 22)). Several dissimilarities exist between RA and experimental rodent models of this illness that can explain the different impact on circulating adipokines. One of them is that experimental arthritis dramatically decreases WAT mass, whereas rheumatoid cachexia is usually associated with increased adipose tissue mass (23). Therefore, it is not surprising that hormones released by adipose tissue are modified differently in both types of arthritis. Another difference is that whereas most RA patients have increased basal plasma insulin levels and insulin resistance (24), arthritic rats have lower serum insulin levels. In this sense, several factors account for the increased incidence of insulin resistance in RA that are not shared by experimental arthritis models such as glucocorticoid therapy, abdominal obesity, and antihypertensive therapy (24, 25). Nevertheless, muscle wasting in chronic inflammatory illnesses can be independent of obesity and insulin resistance. It has recently been reported in chronic obstructive pulmonary disease that obese patients showed insulin resistance, whereas cachectic patients remain insulin sensitive (26). In mice with cancer cachexia, the PPARγ (PPARG) agonist rosiglitazone, an insulin sensitizer, increases adipose tissue but not muscle mass in late-stage cachexia (27).

The dramatic decrease in *Pparα* expression in soleus by arthritis is in accordance with our previous data (11), in which gastrocnemius *Pparα* expression is decreased in arthritic rats and normalized by fenofibrate treatment. The decrease in *Pparα* mRNA during inflammation can be due to a direct effect on myogenic cells, as cytokines are able to decrease *Pparα* mRNA levels (28). Inflammation decreases fatty acid uptake and oxidation in skeletal muscle, shifting its metabolism from fatty acids to glucose as preferred fuel substrate (29). PPARα deficiency results in a defect in fatty acid uptake and oxidation (30). Therefore, the arthritis-induced decrease in *Pparα* could also contribute to decrease in fatty acid oxidation and to muscle disturbances, whereas its normalization by fenofibrate treatment may modulate soleus mass recovery. In contrast, fenofibrate was not able to increase WAT weights in arthritic rats. These data can be explained by the fact that fenofibrate facilitates the mobilization of lipid depots as fuel (30) and lowers adiposity (31). Similarly, fenofibrate was unable to normalize serum insulin or liver glycogen. Furthermore, in control rats, fenofibrate decreased liver glycogen. Inhibition of liver gluconeogenesis, without changes in muscle glucose metabolism after fenofibrate, has been reported (32). These authors proposed that fenofibrate may have a favorable effect on glucose metabolism by inhibiting gluconeogenesis in the liver and maintaining systemic lipid and insulin-dependent muscle glucose uptake.

It has been reported that arthritic rats have reduced adipocyte size and downregulated membrane glucose transporter type 4 (33). Leptin levels directly correlate with WAT mass, adipocyte size, and caloric intake (12). Taking into account that pair-fed rats have lower leptin levels than control rats and that serum concentration of leptin in arthritic rats is half of that observed in pair-fed rats, the inhibitory effect of arthritis on leptin levels seems to be mostly due to inflammation, rather than the decrease in food intake. The increased leptin levels in arthritic rats that received fenofibrate do not seem to be due to an increase in fat mass, as fenofibrate did not increase fat weight. A stimulatory effect of fenofibrate on leptin release can be excluded, as fenofibrate did not increase leptin levels in control rats. Furthermore, fenofibrate decreases leptin levels in animal models of obesity (34, 35, 36) and decreases leptin secretion by adipose cell cultures from dyslipidemic but not from normolipidemic humans (37). Therefore, the stimulatory effect of fenofibrate on leptin levels in arthritic rats can be related to the improvement of metabolic state.

Arthritis-induced decrease in adiponectin levels does not seem to be secondary to the decrease in food intake; on the contrary, caloric restriction increases adiponectin levels (38). Taking into account that this adipokine has been shown to be downregulated by cytokines such as TNF and IL6 (reviewed in (39)), arthritis-induced decrease in adiponectin may be due to the inflammatory mediators. In this sense, administration of the anti-inflammatory drug meloxicam, a cyclooxygenase-2 inhibitor, increases adiponectin in arthritic rats (40). Although, mentioned earlier, adiponectin has been reported to be increased in RA patients, an increase in adiponectin levels after treatment with antirheumatic drugs in patients with RA (41, 42, 43, 44) or after anti-TNF therapy in psoriatic arthritis patients (45) has also been described. Fenofibrate also raises adiponectin levels in patients with metabolic syndrome, and this increase is associated with a decrease in multiple inflammatory markers (46), and with a reduction of insulin resistance (47). Accordingly, it is possible that fenofibrate treatment increases serum adiponectin in arthritic rats as a result of its anti-inflammatory effect. Another possibility is that PPARα stimulation increases adiponectin in adipose tissue, as a direct stimulatory effect of fenofibrate on adiponectin has been reported in adipocyte cell cultures (37, 48).

Arthritis-induced skeletal muscle wasting seems to be mostly due to inflammation (4). Serum levels of adiponectin were found to be positively correlated with the improved skeletal muscle mass. It is possible that the ability of adiponectin to stimulate glucose transport and fatty acid oxidation in skeletal muscle (49) and to decrease inflammation (12) contributes to the amelioration of soleus mass in arthritic rats treated with fenofibrate. The protective effect of fenofibrate against arthritis-induced decrease in muscle weight is higher in soleus than in gastrocnemius. As we have reported in the gastrocnemius muscle (11), fenofibrate prevented upregulation of *Tnf* and *Murf1* and also decreased myostatin expression in soleus. However, fenofibrate decreased myogenin and *MyoD* expression in the gastrocnemius of arthritic rats (11) and it increased both myogenic regulatory factors in soleus.

MyoD and myogenin are considered to be key regulatory molecules in early muscle differentiation, but expression continues in mature muscle tissue of adult animals, suggesting that they may play a more extended role. Several studies suggest that these myogenic regulatory factors are involved in regulating the metabolic processes intrinsic to muscle catabolism or anabolism (17, 50, 51). Furthermore, myogenin expression has been reported to play an important role in muscle metabolism and is associated with oxidative enzymes (50, 51). Overexpression of myogenin in muscle cells increases oxidative enzymes, while it reduces glycolytic ones (50). Therefore, the effect of fenofibrate on myogenin and *MyoD* expressions can contribute to the higher anticachetic effect of fenofibrate on soleus than on gastrocnemius.

In conclusion, our data indicate that administration of fenofibrate, a hypolipidemic agent, prevents the inhibitory effect of chronic arthritis on soleus mass. This effect is associated with an increase in circulating adiponectin and upregulation of muscle *MyoD* and myogenin expressions.

## Figures and Tables

**Table 1:** Primers for real-time PCR.

**Gene**	**Forward primer** (5′–3′)	**Reverse primer** (5′–3′)	**Product size** (bp)
*18s*	GGTGCATGGCCGTTCTTA	TCGTTCGTTATCGGAATTAACC	60
*Ppar* *α*	CAACAGCCACTGGTGCAT	TAGGGCCACGTGTAGCAAG	93
*Tnf*	GCCACCAGCTCTTCTGTCT	GTCTGGGCCATGGAACTGAT	100
*ATROGIN1*	GAACAGCAAAACCAAAACTCAGTA	GCTCCTTAGTACTCCCTTTGTGAA	74
*Murf1*	TGTCTGGAGGTCGTTTCCG	ATGCCGGTCCATGATCACTT	58
Myostatin	TGGGCATGATCTTGCTGTAA	TGTTACTTTGACTTCTAAAAAGGGATT	76
Myogenin	CCTTGCTCAGCTCCCTCA	TGGGAGTTGCATTCACTGG	94
*MyoD*	GGAGACATCCTCAAGCGATGC	AGCACCTGGTAAATCGGATTG	104

**Table 2:** Effect of arthritis and fenofibrate treatment on body, epididymal white adipose tissue (WAT), gastrocnemius and soleus weights, as well as on concentrations of serum glucose and liver glycogen. Fenofibrate (feno) (300 mg/kg in 500 μl of 1% carboxymethyl cellulose) or vehicle (500 μl of 1% carboxymethyl cellulose) was administered by oral gavage daily, from days 4 to 15 after adjuvant injection. Data represent mean±s.e.m. (*n*=16–18 rats).

	**C**	**C** **+** **feno**	**AA**	**AA** **+** **feno**	**PF**
Body weight (g)					
Day 4	152±3.4	154±3.4	152±2	152±2.3	149±3.8
Day 15	219±3.6	226±4.3	172±3.2*^,^^∥^	186±6^¶^^,^^‡^	185±4.8*
Gain	67±1.9	72±2.3	20±3.3*^,^^§^	34±1.6 ^¶^^,^^†^	36±1.4*
Epididymal WAT (mg)	1307±104	1186±79	464±49*^,^^§^	640±78^¶^	814±38*
Gastrocnemius (mg)	1127±43	1053±31	555±38*^,^^§^	753±32 ^¶^^,^^†^^,^^§^	929±27*
Soleus (mg)	83±3	87±4	48±2.3*^,^^§^	77±5.2^†^	72±2.8
Glucose (ng/dl)	132±3.9	132±3.8	122±3.2	130±4.7	123±4.1
Liver glycogen (mg/g)	31.1±1.4	19.1±1.1*	8.9±1.7*^,^^§^	7.1±1.4^¶^^,^^§^	26±3.0

C, control rats; AA, arthritic rats; PF, pair-fed rats. *P<0.01 vs control rats, ^†^ *P*<0.01, ^‡^ *P*<0.05 vs arthritic rats treated with vehicle, ^§^ *P*<0.01, ^∥^ *P*<0.05 vs pair-fed rats, ^¶^ *P*<0.01 vs control rats treated with fenofibrate.
